# Urinoma: A Rare Complication of Abdominal Aortic Aneurysm Surgery

**DOI:** 10.1100/tsw.2004.48

**Published:** 2004-06-07

**Authors:** Koon-Sung Cheng, Hashim Hashim, Janice Tsui, Caroline Osborne, Nickolas Law

**Affiliations:** Department of Surgery, Chase Farm Hospital, Enfield, UK

## Abstract

Urinoma or para-renal pseudocyst generally occurs as a result of trauma to the pelvi-ureteric system. It consists of an encapsulated collection of extravasated urine and is usually located in the peri-renal space or more uncommonly in the peritoneal, pleural or mediastinal cavities. There is only one previously reported case of urinoma secondary to abdominal aortic aneurysm (AAA) surgery. We report a case of symptomatic urinoma after infra-renal AAA repair and discuss the etiology, diagnosis and treatment of this unusual condition.

## INTRODUCTION

Urinoma or para-renal pseudocyst generally occurs as a result of trauma to the pelvi-ureteric system. It consists of an encapsulated collection of extravasated urine and is usually located in the peri-renal space or more uncommonly in the peritoneal, pleural or mediastinal cavities. There is only one previously reported case of urinoma secondary to abdominal aortic aneurysm (AAA) surgery (1). We report a case of symptomatic urinoma after infra-renal AAA repair and discuss the etiology, diagnosis and treatment of this unusual condition.

## CASE REPORT

A 68 year-old lady underwent an uncomplicated repair of a symptomatic 5.9cm infra-renal AAA with a straight Dacron graft. One week post-operatively, she developed persistent left lower abdominal pain. A contrast CT scan revealed a hematoma in the left flank and reduced uptake of the contrast in the left lower pole of the renal cortex (Figure 1). The hematoma was managed conservatively but five weeks later she complained of further abdominal pain, painless swelling of the left leg and on examination an abdominal mass was palpable. The diagnosis of deep venous thrombosis was excluded by duplex scan. A second contrast CT scan showed a 15 x 13cm mass arising from the left kidney with a fluid level in the superior aspect (Figure 2). An aortogram excluded the presence of a false aneurysm. The diagnosis of an urinoma was made. This was confirmed by the creatinine concentration of 1050mmol/l from the fluid drained via a

*Deceased.

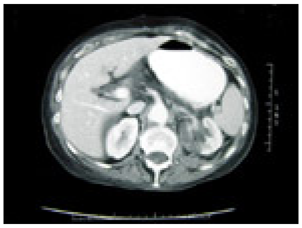

**FIGURE 1.** Contrast CT scan showing the partial uptake of the left kidney.

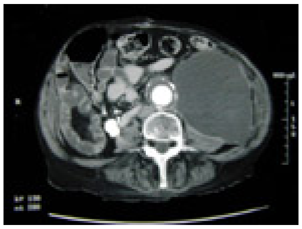

**FIGURE 2.** Contrast CT scan (5 weeks later) showing the large urinoma adjacent to the Dacron graft used for the AAA repair.

percutaneous catheter compared to a plasma creatinine concentration of 108mmol/l and urea of 7.2mmol/l. A ureteric stent was also inserted resulting in the gradual resolution of the mass. Her renal function remained normal at all times.

## CASE REPORT

Urinoma is a well-recognised complication of blunt and penetrating trauma to the pelvi-ureteric system. It has also been reported as the result of 7.5cm iliac artery aneurysm that caused mechanical compression of the pelvi-ureteric system and subsequent calyceal rupture (2) and after hydronephrosis secondary to aorto-bi-iliac graft for AAA (1). This is a case of urinoma occurring after AAA repair, where the aetiology of the condition is either embolisation from an atheromatous aortic wall to the lower pole renal arteries or ligation of the lower pole renal artery leading to necrosis and disruption of the renal pelvis. The appearance of surgical clips in the region of the neck of the AAA supports the latter hypothesis. These clips were applied intra-operatively to control bleeding in this region. A pre-operative CT scan did not show a lower pole vessel that could have been avoided.

The diagnosis of urinoma is suggested by the presence of a "soft tissue mass" on abdominal x-ray. Intravenous urography may show a reduction or absence of contrast excretion from the area. Extravasation of contrast is only seen if the renal function is maintained. Scintigraphy or contrast CT scan is usually diagnostic if ultrasonography is unhelpful (2). CT scanning offers more precision in locating the urinoma in relation to the surrounding structures and is superior in differentiating urinoma from haematoma or abscess collection to other imaging techniques (3). Occasionally, percutaneous or retrograde pyelography is required to define the nature and location of the mass and to aid the percutaneous procedure (3).

Therapeutic intervention for urinoma involves percutaneous catheter drainage with or without ureteric stenting to promote drainage of urine from the kidney into the bladder (4). The latter is required in the presence of ureteric obstruction or injury. Renal function should be closely monitored and ultrasonography or contrast CT should be performed to monitor the resolution of the mass (4). Surgical drainage and repair may be required in some cases, such as delayed hemorrhage, persistent urinoma or hemodynamic instability (2). Open surgery usually results in renal parenchyma loss. Rarely selective renal artery embolisation may be required particularly in the case of traumatic urinoma (5).

It is important during AAA surgery that structures in the region of the neck of the aneurysm are identified to avoid accidental ligation of the lower pole renal artery and that care must be taken when cross-clamping the neck of the AAA to avoid embolisation. Pre-operative angiography or / and CT scan may provide information on lower pole renal artery and the presence of thrombus around the neck of the AAA.
